# Progress in Elucidating Potential Markers and Mechanisms of Rapid Protection Conferred by the VSV-Vectored Ebola Virus Vaccine

**DOI:** 10.1128/mBio.01597-19

**Published:** 2019-07-16

**Authors:** Gary Wong

**Affiliations:** a Institut Pasteur of Shanghai, Chinese Academy of Sciences, Shanghai, China; b Département de Microbiologie-Infectiologie et d'Immunologie, Université Laval, Québec, Québec, Canada

**Keywords:** Ebola, vaccine, vesicular stomatitis virus

## Abstract

Research progress over the past 20 years has yielded several experimental Ebola virus (EBOV) vaccine candidates, which were shown to be effective in nonhuman primates when given 28 days before a lethal challenge. Of these, the vesicular stomatitis virus (VSV)-vectored vaccine against EBOV (VSV-EBOV) is unique at being able to induce rapid protection, with 100% survival achieved as soon as 7 days after EBOV challenge. In a recent *mBio* article, Menicucci et al. carried out a transcriptome analysis of host responses in monkeys immunized with VSV-EBOV from 28 to 3 days before challenge (A. R. Menicucci, A. Jankeel, H. Feldmann, A. Marzi, and I. Messaoudi, mBio 10:e00597-19, 2019, https://doi.org/10.1128/mBio.00597-19). It was found that surviving animals had a controlled innate immune response coupled with rapid adaptive immunity, but this was not detected in nonsurviving animals. These studies highlight the important role innate immunity plays in creating an antiviral state to restrict EBOV replication and ensuring enough time for the vaccine to induce an effective adaptive immune response.

## Abstract (precis)

Research progress over the past 20 years has yielded several experimental Ebola virus (EBOV) vaccine candidates, which were shown to be effective in nonhuman primates when given 28 days before a lethal challenge. Of these, the vesicular stomatitis virus (VSV)-vectored vaccine against EBOV (VSV-EBOV) is unique at being able to induce rapid protection, with 100% survival achieved as soon as 7 days after EBOV challenge.

## TEXT

Ebola virus (EBOV), which causes sporadic and unpredictable outbreaks in Central and West Africa, is one of the deadliest pathogens known to humans. After exposure to the virus, there is an asymptomatic incubation period of 2 to 21 days before the onset of symptoms. The disease course itself is rapid and includes hypovolemic shock, hemorrhage, and multiorgan failure, with death typically occurring at 7 to 10 days after symptom onset. Case fatality rates can reach up to 90%, and there are no specific vaccines or therapeutics licensed for use against EBOV. Due to its rapid pathogenesis, fast-acting countermeasures must be deployed against EBOV infections in order to have any chance for success.

Over the past 2 decades, several vaccine candidates have been tested and shown to be efficacious in nonhuman primates (NHPs) against a lethal EBOV challenge (reviewed in reference 1). One promising candidate is the recombinant vesicular stomatitis virus (VSV)-vectored vaccine expressing the EBOV glycoprotein (VSV-EBOV), which was developed by scientists at the Public Health Agency of Canada during the 2000s. VSV-EBOV is unique in that a single intramuscular injection of this live, replication-competent vaccine can provide rapid immunity leading to partial and complete protection within 3 and 7 days of vaccination, respectively, in NHPs (2). In addition, administration of VSV-EBOV at 30 min postexposure also yields 50% protection in the NHP model (3).

Due to these properties, VSV-EBOV was the countermeasure of choice given to laboratory workers in case of accidental exposure when working with live EBOV (especially before the development of EBOV glycoprotein-specific monoclonal antibody cocktails, such as ZMapp). In 2009, VSV-EBOV was administered to a researcher who had a high-risk occupational EBOV exposure in Germany, at 48 h after the incident, and in 2014 the vaccine was given to a physician working at an EBOV treatment center in Sierra Leone, at 43 h after the needlestick injury. Aside from mild symptoms, including fever at 12 h after the vaccine injection, both recipients remained healthy.

Additionally, VSV-EBOV has been deployed for use on compassionate grounds during outbreak and epidemic scenarios, especially with ring vaccination of high-risk contacts to laboratory-confirmed EBOV cases, as well as contacts to these high-risk contacts. Indeed, several phase I to III clinical trials were initiated during the 2013 to 2016 EBOV epidemic in West Africa: Partnership for Research on Ebola Vaccines in Liberia I (PREVAIL I; NCT02344407), Sierra Leone Trial to Introduce a Vaccine Against Ebola (STRIVE; NCT02378753), and Ebola ça suffit! in Guinea (PACTR201503001057193). Importantly, the Ebola ça suffit! clinical trial had an estimated vaccine efficacy of 100% (4). Preliminary findings from a ring immunization trial initiated during the current 2018 to 2019 EBOV outbreak in the Democratic Republic of the Congo yielded an estimated efficacy of 97.5% over ∼91,000 vaccinees. Importantly, there were no deaths reported among vaccine recipients who developed EBOV disease at ≥10 days after immunization (5).

It is well established that adaptive immune responses (especially humoral immunity) play a major role in VSV-EBOV-mediated protection against EBOV infection, and past studies have highlighted the role of EBOV-specific IgG in statistically correlating with survival of EBOV disease (6) as well as playing a critical role in protection against lethal challenge in NHPs (7). These results provide a reliable, easily measured specific marker for predicting protection induced by VSV-vectored vaccines once adaptive immunity has been established. However, the mechanism(s) for how VSV-EBOV provides rapid (and postexposure) protection against EBOV infection is not well characterized.

Menicucci et al. addressed this knowledge gap in a study (8) published in a recent issue of *mBio*. The authors undertook a thorough and detailed analysis of the transcriptome response in the whole blood of groups of 2 to 3 NHPs immunized with VSV-EBOV and then challenged with EBOV at various times after vaccination (3, 7, 14, 21, or 28 days). Analysis of how gene expression changed after immunization showed the detection of 83 and 518 differentially expressed genes (DEGs) at days 3 and 7 postvaccination, but very few DEGs from day 14 onwards. The majority of DEGs from days 3 and 7 were found to be interferon-stimulated genes (ISGs) important for antiviral defense, and those exclusively identified at day 7 play a role in antiviral innate immunity, including transcription factor genes *STAT1* and *IRF1*, inflammatory mediator genes *MYD88*, *NFKBIA*, and *IL1B*, as well as pathogen recognition receptor genes such as *TLR2* and *TLR4*. Additionally, the authors also detected increased expression of genes important for antigen presentation (*HLA-DQB1*, *CD83*, and *CD1C*), T-cell signaling and activation (*CD2*, *CD4*, *CD28*, *LAT*, *TRBV2*, and *ZAP70*), as well as B-cell signaling, activation, proliferation, and differentiation into plasma cells (*CD19*, *SLAMF1*, *CD40LG*, *IGHA1*, *LYN*, *BAFF*, and *BLIMP1*) from day 7 samples. These findings are supported by the detection of elevated levels of alpha interferon (IFN-α) from NHP samples collected on days 3 and 7, as well as robust antibody responses detected from day 14 onwards. Thus, VSV-EBOV induces a robust innate immune immunity and an early adaptive response within 7 days postvaccination.

**FIG 1:**
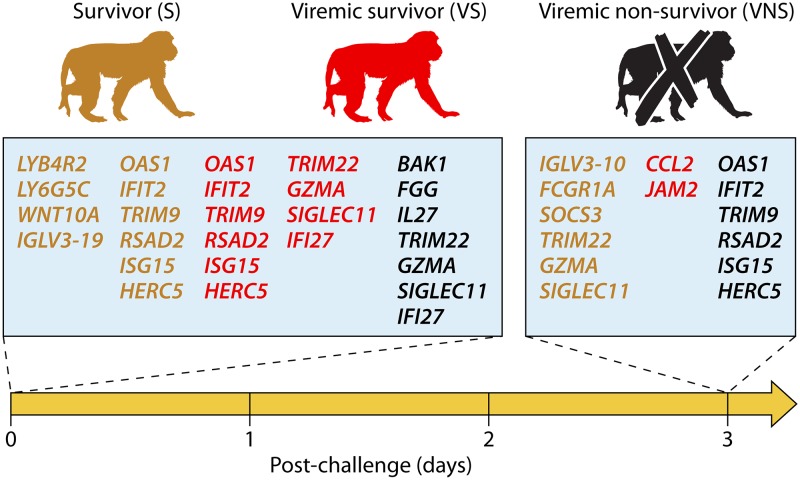
Early markers detected in nonviremic survivor (S), viremic survivor (VS), and viremic nonsurvivor (VNS) after Ebola virus infection. Nonhuman primate results are colored based on survivor outcome: S, brown; VS, red; VNS, black. Detected genes related to the immune response were also color coded accordingly, depending on the outcome group.

The authors then provided data on NHPs challenged with EBOV at 3 days postvaccination with distinct survival outcomes: viremic nonsurvivor (VNS [*n* = 1]), viremic survivor (VS [*n* = 1]), and nonviremic survivor (S [*n* = 1]), thus allowing for comparison of markers between animals with different survival outcomes and potentially a gene signature determining vaccine efficacy and protection. The data showed that genes involved in innate immunity (*LTBAR2*, *LY6G5C*, and *WNT10A*) and humoral immunity (*IFLV3-19*) were highly expressed in S at the day or challenge, compared to VS and VNS, but were reduced by 3 days after challenge (dpc), indicating a controlled innate immunity. ISGs (*OAS1*, *IFIT2*, *TRIM9*, *RSAD2*, *ISG15*, and *HERC5*) were already highly expressed in S and VS at the day of challenge, but this increase in expression was delayed in the VNS animal until 3 dpc. In contrast, genes upregulated in the VNS animal only at the day of challenge include *BAK1* (involved with apoptosis), *FGG* (coagulation), and *IL-27* (which promotes anti-inflammatory cytokine interleukin-10 [IL-10]), among others (Fig. 1). Thus, these results suggest that the presence of a regulated innate immune response coupled with rapid adaptive immunity (such as EBOV-specific antibodies) contribute to protection from EBOV, whereas the absence of early innate immunity combined with vascular disease is indicative of a fatal outcome. These results are consistent with and support the data from a previous study showing that in VSV-EBOV-immunized humans, IFN-γ-induced protein 10 (IP-10), macrophage inflammatory protein 1β (MIP-1β), and monocyte chemoattractant protein 1 (MCP-1) were significantly induced by 1 day postvaccination and that IP-10 levels were positively correlated with EBOV-specific IgG responses (9).

This work provides an in-depth characterization into the early innate immunity observed after VSV-EBOV vaccination and potential markers to pursue in the search of early immune correlates to survival from EBOV infection. In addition, the data contribute another pillar to the mechanism of immune protection generated by the VSV vaccine platform, which can also be applied to other VSV-vectored vaccines beyond EBOV. It will therefore be important to delineate clearly the relative contribution of the innate response versus the rapid early adaptive response toward survival. Since markers indicating early adaptive immunity could be detected in these VSV-EBOV experimental results (and antibody responses can be detected sometimes as early as 5 days after immunization), a potential follow-up study could involve work with a nonrelated VSV vaccine (such as those against Marburg or Lassa viruses) with larger groups of NHPs, such that the role(s) of the innate immune response markers can be clearly defined and supported by statistics. This study is an important building block for the elucidation and characterization of early gene signatures contributing to survival from EBOV and will be of essential reference when designing future milestone studies regarding the mechanisms of nonspecific protection elicited by VSV-vectored vaccines.
